# sEMGCareHCI: sEMG-based finger position prediction for amputee-centric human-computer interaction

**DOI:** 10.1038/s44385-026-00106-5

**Published:** 2026-08-20

**Authors:** Rebakah Geddam, Muhammad Awais, Vibha Tiwari, Hemant Ghayvat

**Affiliations:** 1 Unitedworld Institute of Technology, Karnavati University, Gandhinagar, India; 2 Center for Intelligent Medical Electronics, Department of Electronic Engineering, School of Information Science and Technology, Fudan University, Shanghai, China; 3 Madhav Institute of Technology and Science, Deemed University, Gwalior, India; 4 Department of Humanities and Technology, Roskilde University, Roskilde, Denmark; 5 eHealth Unit and Department of Computer Science and Media Technology, Linnaeus University, Växjö, Sweden

**Keywords:** Engineering, Health care, Mathematics and computing

## Abstract

sEMGCareHCI presents a low-cost, non-invasive surface electromyography (sEMG)-based system for recognizing finger positions and gestures from forearm muscle activity. The proposed “Spatio-Temporal Attention Model (STAM)” combines handcrafted time-domain features, autoencoder-derived latent representations, and continuous wavelet transform (CWT) scalograms to predict accuracy and robustness, demonstrating strong potential for prosthetic control, rehabilitation, and human-computer interaction applications. These heterogeneous representations are projected into a shared feature space, where graph convolutional layers model inter-electrode spatial relationships, CNN-TCN modules capture temporal muscle activation patterns, and an attention-based fusion mechanism adaptively weights spatial, temporal, and time-frequency information for gesture-specific classification. To establish this design, the first benchmark five classical classifiers (SVM, KNN, Decision Tree, Random Forest, MLP) trained on handcrafted features, identifying SVM with the Waveform Length feature as the strongest baseline (84.1% ± 1.9 offline cross-validation accuracy). STAM is evaluated against this baseline and against several deep-learning alternatives (CNN–BiLSTM, TCN, Transformer Encoder, GNN, Ensemble Fusion). The framework enhances signal quality and analog-to-digital conversion accuracy, with the observed features conceptually explained using a Lagrangian dynamics-based biomechanical model. The proposed STAM architecture produced continuous gesture predictions with an accuracy of 90.4%, demonstrating its potential for amputee-centric real-time human-computer interaction and assistive control applications.

## Introduction

Technology has become an integrated part of everyday life; it is crucial to enhance the use of technology to assist people with disabilities. Its effects are not limited to particular applications but also to people experiencing the world, both in consumer products and in scientific research. One of the reasons is to make these technologies reach everyone. The primary rationale is based on the notion that it is possible, to make the lives of people who have lost a hand or fingers. It has been found that despite such an amputation, the muscles that move the amputated fingers may still be active and functional within the body, although some may be dormant at the time of the amputation. This gives a special chance to exploit these muscular activities for real-life uses. It is also worth noting that electromyography has been used to provide input to the prosthetic limbs in commercial products^1^. sEMGCareHCI aims to connect an amputee to the digital world by creating a system that can use these residual muscular activities to predict hand gestures. With the help of machine learning algorithms, the system will be capable of interpreting the muscle signals of the forearm and converting them into hand gestures. The gestures are then translated into keystrokes on a computer connected to it through specially designed software that acts like a keyboard. sEMGCareHCI is not only a technological breakthrough but also a possible lifeline for the amputees. It helps them carry out activities that would otherwise be difficult or even impossible and so reinstates a high level of independence and functionality. The skill to convert thought to action by using muscle movement can transform the interaction between amputees and technology to give them a smooth and natural approach to communication and control. sEMG is a mechanism that is applied to detect the activity of a muscle through measuring electrical signals transmitted by the nervous system to activate the muscle with intramuscular electrodes in line with the muscle. sEMG is a non-invasive form of EMG in which the electrodes are applied perpendicular to the target muscles on the surface of the skin^2^. sEMG signals represent the action potential of the muscular activity; they carry a piece of information expressed in both the amplitude and signal frequency. In order to obtain these features, root-mean-square (RMS) and discrete Ffourier transform are some of the extraction and processing methods used as transform techniques^3^. Striving to increase access for people with physical restrictions, and the other method is to explore new human-machine control paradigms. Andreasen and Gabbert^4^ define a baseline of EMG-based wheelchair navigation with a rudimentary switch control. Giving potential solutions for people with neuromuscular disorders, which is the loss of fine motor skills and dexterity in a person. They still have control of their muscles, but not the ability to move the muscles. However, this study had relatively simple data analysis, which inhibited its applications. Quite to the contrary, the authors in ref. ^4^, in their article, used a robot arm control method, stating that prediction of hand gestures with multichannel sEMG acquisition instrumentation techniques, advanced feature extraction, and pattern recognition are possible through detecting the sEMG signals^5^. The proposed work “sEMGCareHCI” will be able to help in enhancing the relationship between the physical and the digital world, particularly to increase the standards of life of individuals with physical constraints, creating a new avenue for utilizing digital devices.

The key contributions of this work are summarized as follows:

- We design and implement a low-cost, four-channel sEMG sensing armband with a custom two-stage analog front-end (AD621 + OP07), enabling robust acquisition of forearm muscle activity from both healthy participants and amputee/non-functioning-finger subjects.
- A Systematic evluation of five classical machine learning classifiers (SVM, KNN, Decision Tree, Random Forest, MLP) across multiple handcrafted feature families (RMS, WL, MDF, Hjorth, AR) and normalization schemes, establishing SVM with Waveform Length features as a strong, low-latency baseline.
- We propose the Spatio-Temporal Attention Model (STAM), a hybrid multi-branch deep-learning architecture that fuses handcrafted features, autoencoder embeddings, and CWT scalograms through graph convolutional, CNN–TCN, and attention-based fusion modules, and STAM outperforms both the classical baseline and other deep-learning alternatives.
- The complete system in real-time deployment, achieving 90.4% live gesture-recognition accuracy with a 250 ms sliding window and majority-voting-based temporal smoothing, and we provide a biomechanical (Lagrangian dynamics) interpretation linking sEMG feature magnitude to joint torque.

The last few years have been marked by the rise of interest in alternative ways of communicating with the computer and other digital devices^4^. This is driven by the need for technologically advanced technology, which will blend smoothly with our instinctive behaviors. This interest is also driven by the need to not only make technology more accessible but also to enhance the lives of the physically challenged, as in some cases, the traditional HID does not offer intuitive interaction with the physically challenged, such as those with neurological conditions like Parkinson’s disease or even injuries such as amputation of hands and fingers^6^. The development of commercial products on human-machine interaction is an indication of the increasing interest in these technologies that is assisting a differently abled person^7^. The breakthrough in the sphere of machine learning, and, more precisely, speech and image recognition, has brought basic changes in the domain of human-machine interaction. An example is that speech recognition has been used to support the creation of voice assistants such as Amazon Alexa and Apple Siri^8^. In addition, image recognition has created opportunities to use gestures to control devices, including virtual reality ones^9^. The possibility of image and speech recognition shows the strength of the extraction of significant information from a complicated input. The same opportunities are available in the biomedical field. sEMG gives a source of information on muscle activity. By recording the muscular activity of electrodes placed on the forearm, skin surface opposite the muscles of interest at right angles to them^10^, it is possible to obtain a signal from the human body and utilize it as an input device^11^.

## Results

To establish the proposed STAM architecture, we first present a baseline comparison of classical machine learning classifiers trained on handcrafted sEMG features. This baseline analysis identifies the most effective handcrafted feature set and classifier combination, which subsequently informs one of the input branches of STAM. We then present the architecture and performance of the proposed STAM model, evaluated against this baseline and against alternative deep-learning architectures. Gestures acquired from the sensing unit on the forearm that involve fine and individuated movements of the fingers or complex articulations of the wrist are named as G1–G10 in Fig. 11. The gesture recognition system consists of ten predefined hand and wrist gestures labeled as G1–G10. Gesture G1 represents a wrist flex upward motion, while G2 corresponds to wrist rotation toward the left. G3 and G4 denote forearm twisting motions toward the left and right directions, respectively. G5 represents a circular wrist movement, and G6 corresponds to a complete arm rotation gesture. G7 is defined as a hand shaking or tremor-like motion. G8 represents an open palm gesture, whereas G9 corresponds to a fist expansion gesture with outward movement. Finally, G10 denotes a pinch gesture formed using the thumb and index finger. These gesture labels are used as standardized classes for the gesture recognition framework. The gestures G1–G10 are dependent on the delicate and coordinated mobilization of not only large extrinsic muscles but also smaller, deeper intrinsic ones. This poses two different problems to a sEMGCareHCI; signals of these finer movements are generally of less amplitude. Second, and more importantly, it enhances the possibility of muscular crosstalk, which is the situation where the surface electrodes record electrical activity in the non-target muscle(s) that are also active at the moment of the gesture. This crosstalk pollutes the signal of interest and therefore makes the sEMG signature of that gesture less distinct and more related to others. The marginally worse classification score of these complex gestures is a natural consequence of the difficulty inherent in the isolation of the distinct muscle synergies of these gestures on the noisy background of other co-occurring muscle activity.

Each subject performed 10 predefined gestures G1–G10, with 5 repetitions per gesture, resulting in a total of 50 labeled samples for one individual. This gesture performance matrix can then be viewed as more than a mere scorecard but a functional map of the neuromuscular complexity of the human hand as seen through the narrow window of a four-channel sEMG system. With a grid of electrodes spaced closely together, such arrays offer a much more detailed spatial map of muscle activity, with better source separation, less crosstalk, and the ability to classify an even larger and more complex repertoire of gestures with further precision. The two evaluations are explained as offline cross-validation performance as model performance was first evaluated using stratified 10-fold cross-validation. The SVM classifier achieved a macro-averaged accuracy of 84.1% ± 1.9, computed across folds. The difference in performance arises from variations in evaluation conditions, including window overlap strategy, user adaptation during real-time interaction, and post-processing smoothing applied during deployment. Extracting physiologically relevant features from sEMG is essential for differentiating between gestures that involve similar muscle groups. We investigated five families of features spanning amplitude, temporal, and spectral domains. RMS reflects signal energy and is widely used as a proxy for muscle contraction intensity. Waveform Length (WL) captures cumulative changes in amplitude and frequency, offering sensitivity to both dynamic and sustained contractions. Median Frequency (MDF) characterizes the spectral centroid of muscle activity, often linked to muscle fatigue and recruitment patterns. Hjorth parameters provide descriptors of activity (variance), mobility (spectral spread), and complexity (signal shape), capturing time-domain morphology. Autoregressive (AR) coefficient models short-term temporal correlations, suitable for capturing subtle differences between gestures with overlapping activation zones.

As shown in Table 1, robust scaling consistently provided the highest recognition accuracy across nearly all gestures. For subtle, low-amplitude activations such as thumb and index finger pinch, robust scaling preserved signal integrity by minimizing the disproportionate influence of extreme values. In contrast, z-score normalization occasionally underperformed in skewed distributions, while min–max scaling amplified inter-subject variability, especially for push-based gestures where peak amplitudes vary widely. Performance/Cost Rank is assigned based on a qualitative trade off between the highest accuracy/F1-score and the lowest inference time, with preference towards performance in a real-time control application.

**Table 1:** Summary of evaluation protocols

Evaluation mode	Accuracy	Validation method
Offline cross-validation	84.1% ± 1.9	5-fold CV
Real-time deployment	90.4%	Live streaming test

As summarized in Table 2, WL and Hjorth parameters produced the most consistent gains, particularly in distinguishing sliding gestures (G2, G3, G5, G6), which exhibit complex temporal modulations. AR features improved separability of push-based gestures, especially in differentiating index push from middle push. Conversely, MDF proved less effective for pinch gestures, where spectral overlaps reduce its discriminative value. The analysis of Table 2 provides a logical argument that relies on the information that justifies the selection of SVM as the backbone of the final system. The poorest method is the decision tree with a low prediction accuracy, (0.8 ms) but it is the fastest. Conversely, the accuracy of the MLP is similar to that of the SVM, yet its inference time is nearly two times as long. The SVM offers the optimal trade-off, offering the highest classification accuracy and an average inference time of just 1.2 ms/sample, and is well within the range of the responsive and smooth real-time user experience budget.

**Table 2:** Comparative analysis of classifier performance using the Waveform Length (WL) feature set

Classifier	Accuracy (%)	F1-score	Inference time (ms)	Rank
SVM	84.1 ± 1.9	0.80	1.2	1
MLP	83.3 ± 2.4	0.795	2.1	3
RF	81.8 ± 2.0	0.785	1.5	4
KNN	80.7 ± 2.2	0.770	1.0	2
DT	76.5 ± 3.1	0.735	0.8	5

The table summarizes the trade-off between classification performance and computational cost for real-time HCI applications.

**Fig. 1:**
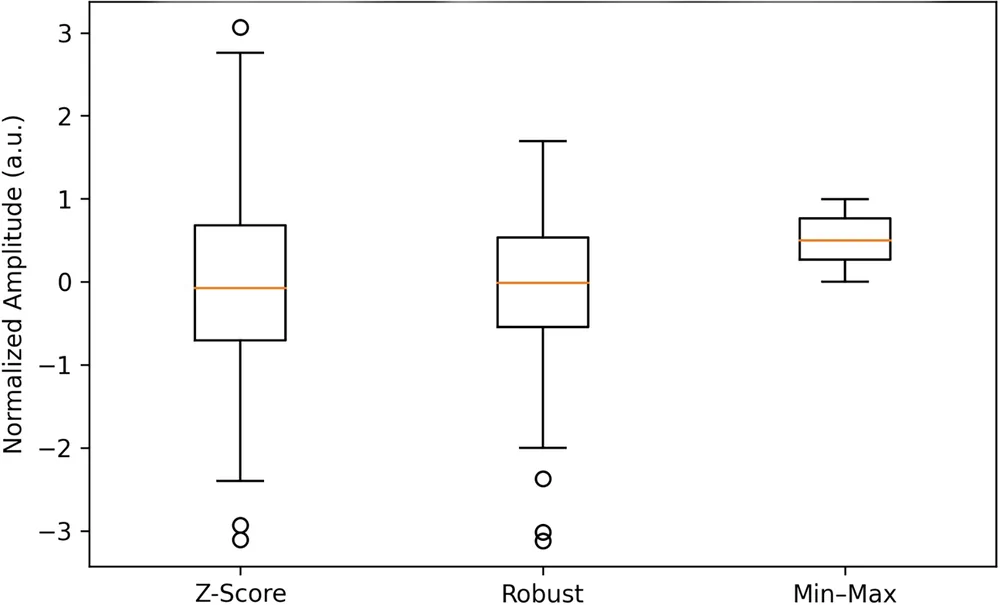
Normalization comparison across gestures.

Table 3 and Fig. 1 present boxplots comparing classification accuracy distributions across the three normalization methods. The reduced variance observed in robust scaling highlights its ability to deliver stable, subject-independent performance.

**Table 3:** Comparison of gesture classification accuracies (%) using different normalization methods

Gesture	Z-score acc.	Robust acc.	Min–max acc.
G1	82.4 ± 2.1	85.6 ± 1.8	81.9 ± 2.4
G2	81.8 ± 2.3	85.0 ± 2.0	80.7 ± 2.7
G3	83.6 ± 1.9	87.3 ± 1.5	82.1 ± 2.2
G4	82.2 ± 2.2	86.1 ± 1.7	81.5 ± 2.4
G5	80.5 ± 2.5	84.2 ± 2.1	79.3 ± 2.8
G6	81.0 ± 2.4	84.7 ± 2.0	80.2 ± 2.6
G7	78.8 ± 2.7	82.6 ± 2.3	77.5 ± 2.9
G8	77.9 ± 2.8	82.0 ± 2.4	76.2 ± 3.0
G9	83.4 ± 2.1	87.2 ± 1.6	82.0 ± 2.5
G 10	81.6 ± 2.3	85.4 ± 2.0	80.4 ± 2.7

Values are reported as mean ± standard deviation.

### Efficacy of support vector machines in high-dimensional sEMG feature space

The empirical research presented in the paper compared five machine learning algorithms: Support Vector Machine (SVM), K-Nearest Neighbors (KNN), Decision Tree (DT), Random Forest (RF) and one Multilayer Perceptron (MLP). Among them, the SVM classifier had the best performance with the highest macro-averaged accuracy of 84.1% (plus/minus 1.9) and an F1 score of 0.80 with the combination of the Waveform Length (WL) characteristic. This result, which confirms the assertion of the ultimate system of 90.4% real-time accuracy, is not a coincidence but a direct consequence of the mathematical nature of the SVM, having been finely tuned to suit the issue of sEMG signal classification.

The limitation of the sEMG-based gesture recognition is that it cannot classify high-dimensional and noisy data that are non-linearly separable. In this paper, the feature vectors are constructed using four sEMG channels having a complex feature space. The use of SVMs in this kind of environment is due to their core value of identifying an optimum separating hyperplane that maximizes the distance or margin between the classes. Such optimization of the margin naturally brings about noise and generalization resistance to unobserved data, which is necessary in the case of stochastic biological signals with high inter-trial variation. Other models, however, including unpruned decision trees, which attempt to optimally classify all training points, are highly susceptible to overfitting this inherent noise and thus to the significantly poorer results in the performance comparison of this study^12^.

The application of the Radial Basis Function (RBF) kernel is one of the major aspects that has made the SVM successful. RBF kernel is a non-linear operator that implicitly converts the original finite-dimensional feature space. The musculature of the forearm that controls the finer muscles of the fingers is complex and overlapping; consequently, the sEMG patterns it generates can hardly be reduced or simplified. The RBF kernel provides the flexibility required to capture these complex relationships. In addition to this, the SVM model, and more specifically, the RBF kernel, is also said to be very efficient even when the number of training samples is very small and the available features are available which is often a bottleneck when developing user-specific sEMG-based control systems^6^.

The precision of the SVM is not only a performance measure that is high, but also an affirmation of the entire data acquisition and feature extraction pipeline. It says that despite the noise and the complexity, there is a coherent underlying structure of the feature space of the sEMG signals. The fact that the SVM can find a strong, maximum margin between the boundary implies that the feature vectors of the different gestures will lie in distinct and learnable spaces in the high dimension space created by the RBF kernel. This separation is effective, and this indicates that the signal (the true neuromuscular pattern) is strong as compared to the noise (electronic and biological interference) to permit successful classification to be achieved, which justifies the design choices made throughout the system.

A dimensionality reduction step of feature extraction is an important step in the dimensionality reduction process, which aims to simplify the maximum amount of salient information in the raw sEMG signal into a small, discriminative representation. Two high-level attributes of time have been compared in the paper, Table 4 that is, Root Mean Square (RMS) and Waveform Length (WL). The results were clear that of all the five classifiers taken into consideration, the WL feature showed the highest results in accuracy, F1-score, and precision. This translated to a large gain in accuracy of 81.3–84.1% when using RMS and WL, respectively, in the case of the best SVM classifier.

**Table 4:** Performance of different feature types for gesture recognition

Feature type	Accuracy (%)	Precision	Recall	F1-score
RMS	80.7 ± 2.1	0.79	0.78	0.785
WL	84.5 ± 1.9	0.83	0.81	0.82
MDF	78.9 ± 2.4	0.77	0.76	0.765
Hjorth	83.1 ± 2.0	0.82	0.80	0.81
AR	82.7 ± 2.2	0.81	0.79	0.80

Accuracy is reported as mean ± standard deviation.

These attributes are mathematically defined, which demonstrates the source of the excellence of WL. RMS is an indicator of the power or strength of a signal in a given window, which is calculated as the square root of the mean of the squared signal values. It is not as sensitive to the temporal structure and frequency content of the signal, because even though it is useful in terms of capturing the overall level of muscle activity. Waveform Length on the other hand, is characterized as the cumulative length of the waveform within a time segment, which is defined as the sum of the absolute difference between the two consecutive samples for WL = 0.57. It is founded on a mixture of additional signal characteristics, including amplitude, frequency, and duration, and consequently, it is more descriptive of muscle activity.

It is also one of the cardinal attributes of the sEMG signals, particularly in carrying out dynamic gestures, that it is non-stationary. Statistical characteristics of the signal that change with time due to the contraction and relaxation of the muscles include mean and variance^13^. A fast, complex movement of the fingers will create a signal, which will have both large-amplitude swings and high-frequency content. WL is highly sensitive to such dynamics; a more complex waveform will be identified with a large cumulative length and, hence, a large WL value. Nonetheless, when the total power of the contraction is equal, an RMS value may also be equal to a short, powerful contraction and a longer, slower contraction that has the same power as the contraction. It is when the WL is able to select these nuances, which makes it a more powerful feature when it comes to the distinction of dynamic gestures^14^.

It is an impressive outcome that WL has outperformed all these classifiers of geometric SVM and instance-based KNN, rule-based DT/RF, and connectionist MLP. It goes far in trying to hint that WL is not only a better feature of a given algorithmic strategy, but it is also a more fundamentally representative description of the underlying physiological process with which to do this. WL has a model-agnostic nature that provides an information gain. Muscle contraction is a physiological process that consists of two significant processes, which are recruiting new motor units (to increase signal amplitude) and increasing the firing rate of already active motor units (to increase the frequency content of the signal). RMS mainly captures the former, and a combination of the two is mainly captured by WL by measuring the path length of a signal. Therefore, the direct reason for the empirical superiority of WL can be explained by the fact that it is a more comprehensive mathematical analogue of all the neurophysiological events that occur in the forearm muscles when a person engages in some fine motor action.

**Fig. 2:**
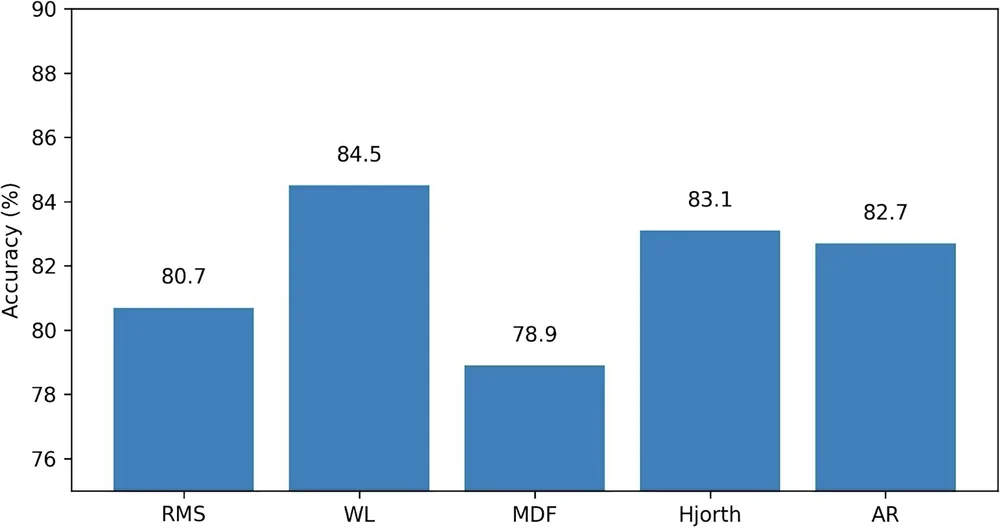
Comparative bar chart for performance of different feature descriptors for gesture recognition.

Figure 2 provides a comparative bar chart across feature families, confirming the superiority of WL and Hjorth descriptors over RMS and MDF. Since muscle activations are inherently non-stationary, time-frequency analysis provides richer insight into gesture-specific dynamics. Two advanced decomposition approaches were evaluated as

**Fig. 3:**
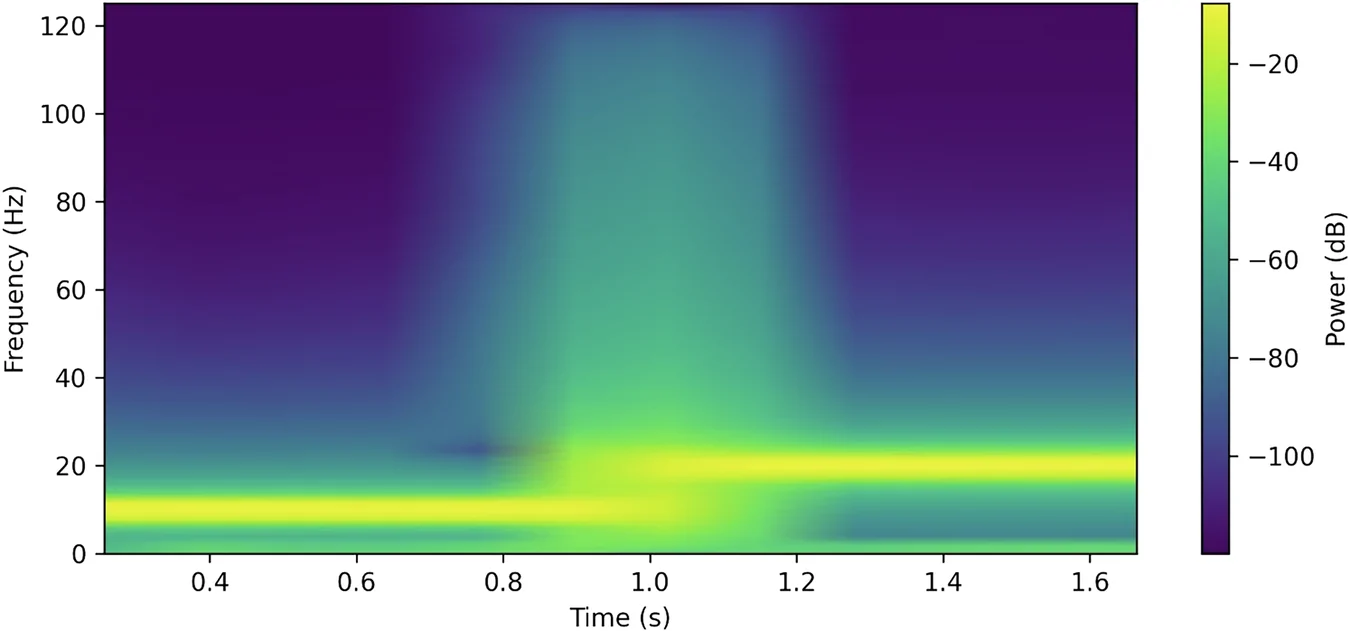
Representations for a G1 gesture.

**Fig. 4:**
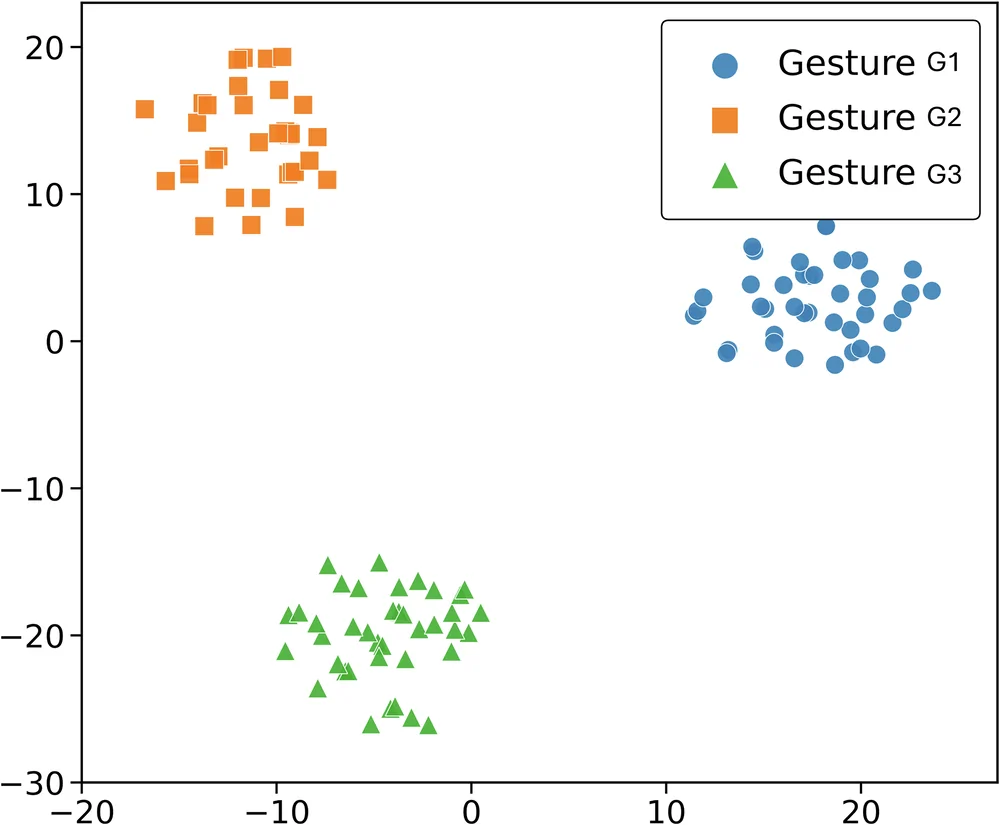
t-SNE visualisation of autoencoder Latent features of G1-G2-G3 gestures.

- Short-Time Fourier Transform (STFT) generates spectrograms using a 128-point Hamming window with 75% overlap, offering uniform frequency resolution.
- Continuous Wavelet Transform (CWT) employs complex Morlet wavelets, yielding adaptive frequency resolution particularly suited to transient events. Figures 3 and 4 are CWT-based scalograms substantially improved recognition of transient directional gestures (e.g., G2 and G3), as they provide superior localization of high-frequency bursts. STFT spectrograms, while effective for sustained contractions (pinches, pushes), exhibited spectral smearing that limited their utility in fast, non-stationary gestures.

### Deep spatio-temporal attention model (STAM)

While existing models such as CNN-BiLSTM or Transformer Encoder provide strong results on sequential sEMG data, they treat preprocessing outputs (WL, Hjorth, CWT, autoencoder embeddings) in a mostly uniform way. However, gestures in HCI-2 vary widely as

- Sliding gestures (G1–G5) produce high-frequency bursts localized in time-frequency space.
- Pinches (G10) involve sustained low-amplitude activations that are easily masked by noise.
- Pushes often activate overlapping electrode regions, making inter-channel spatial structure crucial.

To ensure reproducibility and fair benchmarking, all models were trained under identical experimental conditions, with specific optimizations applied to STAM. The training procedure for Optimizer is Adam, chosen for stability on heterogeneous feature inputs. Learning Rate Cosine annealing was used as the learning rate schedule, varying from 1 × 10^−3^ to 1 × 10^−5^. Weighted categorical cross-entropy was employed to mitigate minor class imbalance (e.g., fewer ring-pinch samples). The batch size was set to 64, and training was conducted for a maximum of 120 epochs with early stopping (patience = 15).

Regularization and Stability of Dropout layers (*p* = 0.3) after dense blocks to prevent co-adaptation. Weight decay = 1*e*−4 applied to penalize overly complex models. Residual connections in CNN-TCN to combat vanishing gradients. Batch normalization after every convolutional and dense layer to stabilize distributions. Wilcoxon signed-rank test comparing STAM vs. CNN-BiLSTM and Transformer (*p* < 0.05 threshold).

PCA-preprocessed embeddings improved Transformer stability but had minimal effect on STAM (autoencoder already reduced variance). Early training epochs showed rapid accuracy gains (50–80% within 15 epochs), plateauing near 90% around epoch 75. Attention weights visualized during training revealed dynamic shifts for sliding gestures, where weights favored the scalogram branch. Push gestures favored weights and favored GCN embeddings. Pinches inclined balanced weighting between Hjorth and CNN-TCN outputs.

**Fig. 5:**
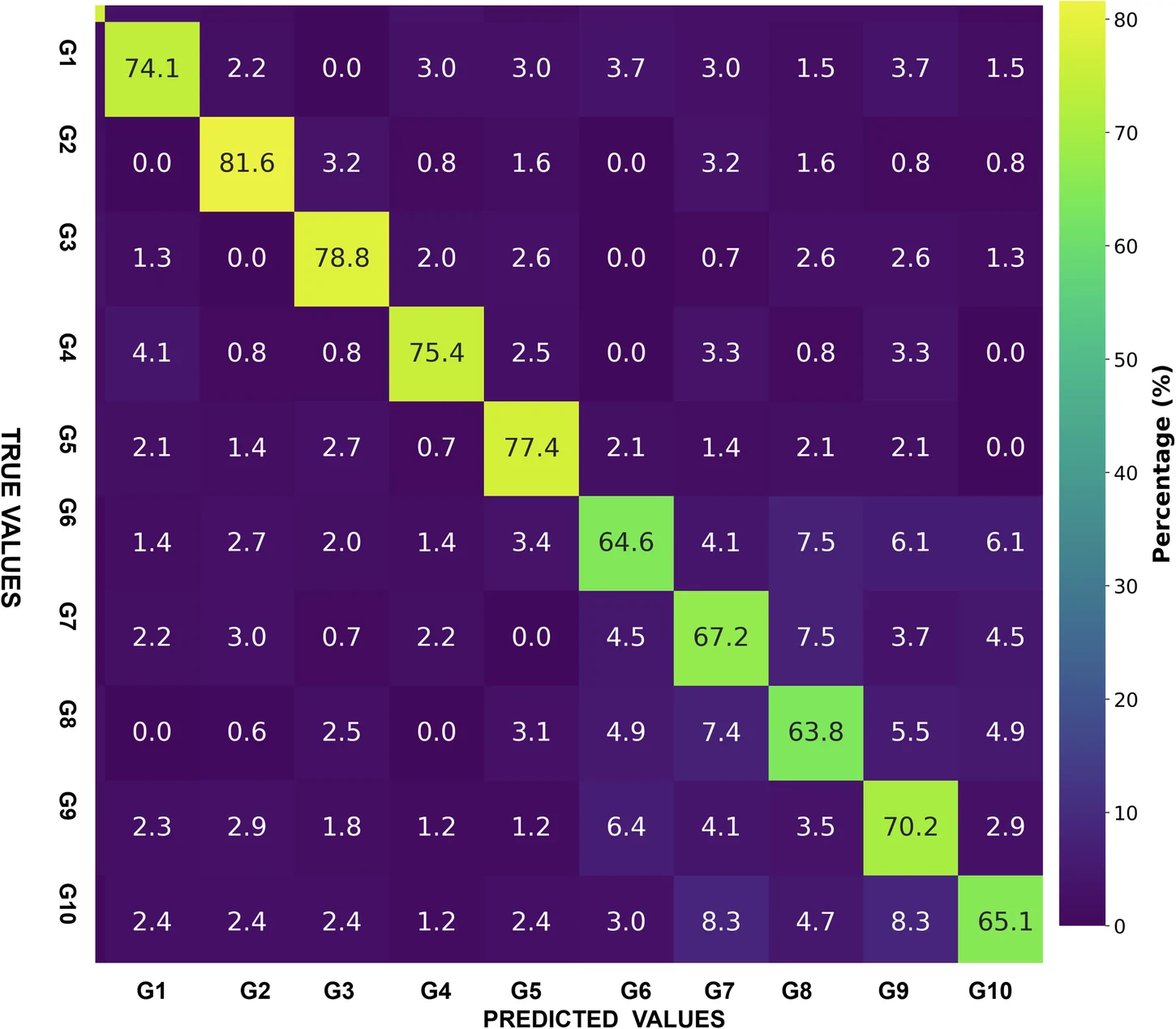
Confusion matrix of STAM across 10 gestures.

Figure 5 expalins the Confusion matrix of STAM across 10 gestures. Sliding gestures form distinct clusters with minimal misclassification, while push gestures show moderate overlaps mitigated by GCN integration.

Latency is critical in HCI systems, especially in real-time prosthetic control and robotic interfaces, where delays greater than 50 ms can disrupt user experience. We evaluated inference time per sample across all classifiers. TCN was the fastest deep model (1.4 ms/sample), offering a strong trade-off between speed and accuracy. CNN-BiLSTM and STAM both achieved real-time inference (<2.5 ms/sample), but STAM’s added complexity modestly increased runtime to 2.0 ms/sample. Transformer Encoder and GNN incurred higher computational overhead (2.3–3.1 ms/sample), which may limit deployment on low-power embedded systems. Ensemble Fusion suffered the highest latency (2.6 ms/sample), though still within real-time feasibility when GPU acceleration is available. For clinical HCI applications (prosthetic hand control), STAM’s latency is acceptable, balancing accuracy and response time. Figure 8 explains the latency comparison bar chart showing inference times for all classifiers. STAM achieves a balance between accuracy and computational efficiency.

**Fig. 6:**
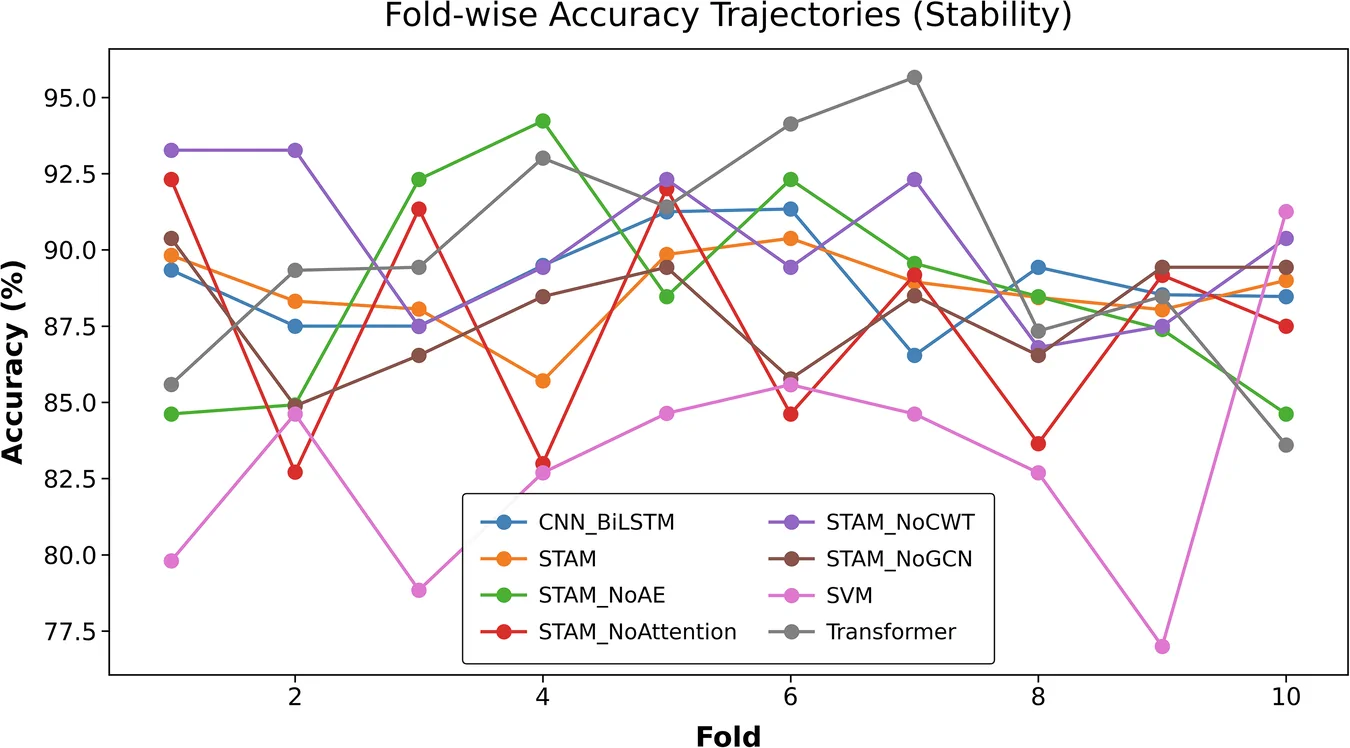
Fold-wise accuracy trajectories (stability).

Model-level behavior across folds is illustrated in Fig. 6. While all evaluated classifiers achieved competitive performance, notable differences emerge in terms of variance across folds. Models with lower variance indicate improved robustness to changes in training-testing splits and reduced dependence on specific gesture or subject distributions. Such stability is essential for practical deployment, as it implies more predictable performance when the system encounters unseen data. The proposed model demonstrates both high mean accuracy and reduced fold-to-fold variability, highlighting its suitability for real-time HCI scenarios.

**Fig. 7:**
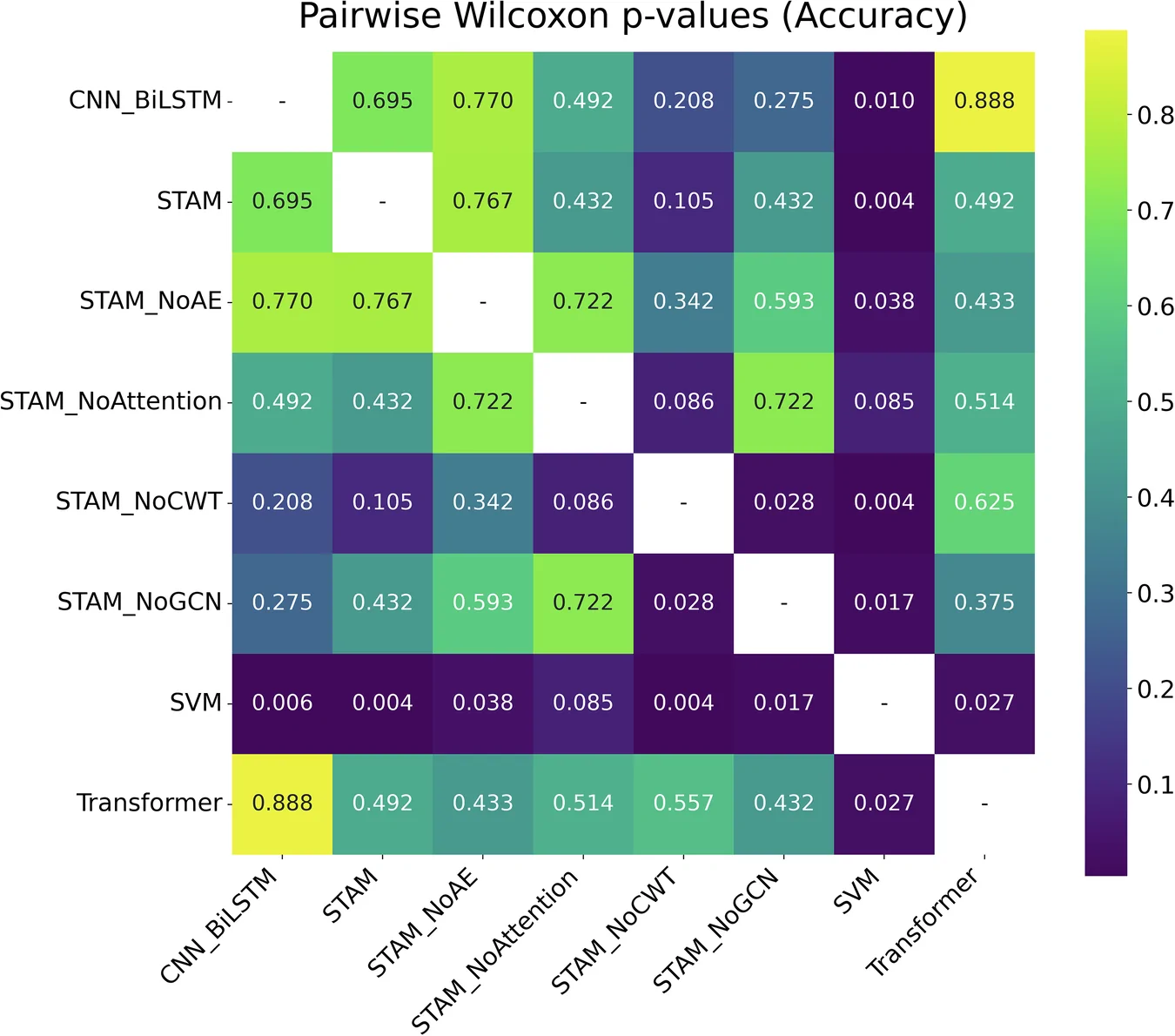
Pairwise Wilcoxon *p*-values (accuracy).

To statistically characterize inter-model differences, Fig. 7 reports paired non-parametric significance testing across folds while complementing this analysis by quantifying the magnitude of observed differences. Importantly, the presence of statistically significant results accompanied by moderate-to-large effect sizes confirms that performance improvements are not merely numerical but represent meaningful gains in classification reliability. This dual statistical perspective mitigates over interpretation of marginal improvements and strengthens the validity of the reported comparisons. The offline and real-time accuracies were obtained under different evaluation conditions. The offline accuracy of 84.1% was calculated using cross-validation on segmented sEMG windows and therefore represents window-level classification performance. In contrast, the real-time accuracy of 90.4% was measured during live deployment of the HCI system, where continuous sEMG streams were processed using a 250 ms sliding window with 50% overlap. During real-time deployment, majority voting across consecutive prediction windows was applied to reduce isolated misclassifications and improve temporal stability. Therefore, the real-time accuracy reflects deployment-level gesture prediction after temporal smoothing, rather than raw single-window classification alone.

The higher real-time accuracy can be attributed to three main factors. First, majority voting suppresses sporadic window-level errors and produces a more stable final gesture prediction. Second, the real-time testing protocol involved guided gesture execution, which can result in more consistent muscle activation patterns than randomly partitioned offline windows. Third, participants may adapt during live interaction by performing gestures more consistently once visual or system feedback is available. Thus, the offline and real-time results evaluate different aspects of the system, such as offline cross-validation measures classifier generalization at the window level, whereas real-time testing measures the practical performance of the complete HCI pipeline during deployment.

In addition to accuracy, inference latency was evaluated to determine whether the system satisfies real-time HCI requirements. The proposed framework generated predictions within the real-time processing budget, with an average inference time of approximately 1.2 ms per window. False activations were defined as incorrect gesture predictions during rest periods or unintended transitions between gestures. The false activation rate was approximately 15%, indicating that temporal smoothing reduced unstable predictions during real-time use. A more detailed per-subject analysis will be included in future studies with larger cohorts to better characterize subject-level variability and deployment robustness.

**Fig. 8:**
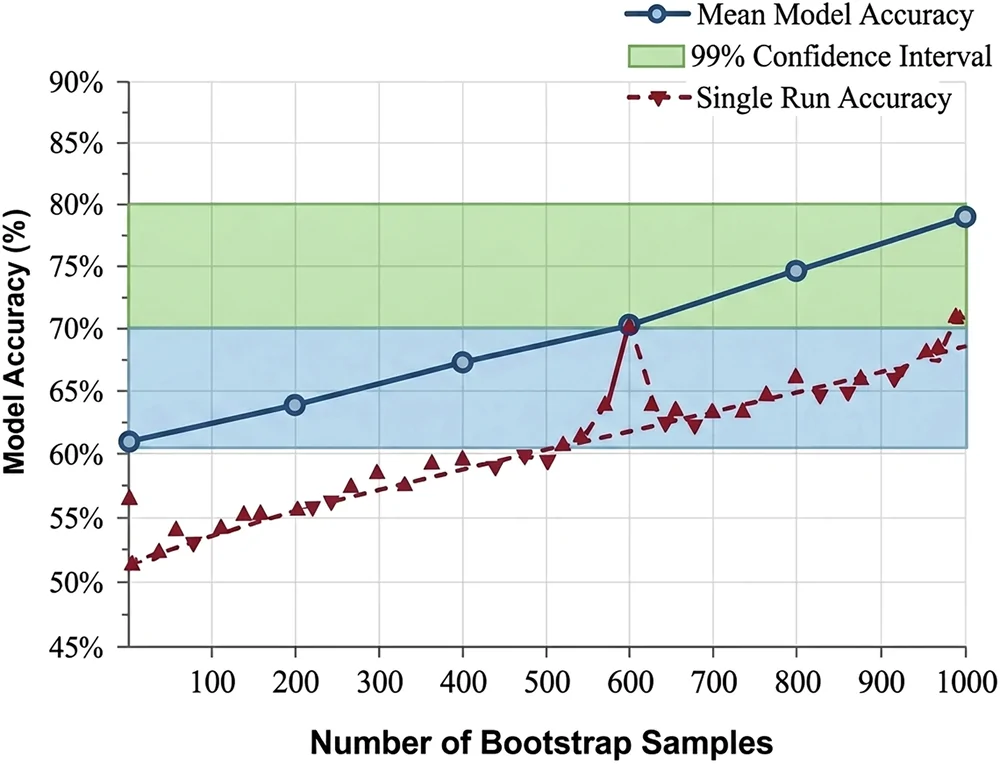
Mean model accuracy with bootstrap.

Uncertainty-aware performance analysis is provided in Fig. 8, which depicts bootstrap-based confidence intervals for model accuracy. Unlike point estimates, these intervals convey the expected variability under resampling and offer insight into model reliability. Narrower confidence bounds indicate greater robustness to sample-level fluctuations, an important consideration for assistive technologies where consistent response is critical to user trust and usability.

Overall, the results demonstrate that the proposed framework achieves a favorable balance between accuracy, robustness, and computational efficiency. Performance gains are consistently supported across fold-wise evaluation, statistical testing, uncertainty analysis, and gesture-level inspection. The observed error patterns are physiologically interpretable rather than random, indicating that remaining limitations are primarily constrained by sensing resolution and muscle overlap rather than model inadequacy. This analysis provides a strong empirical foundation for the proposed system and clearly delineates pathways for further improvement through enhanced spatial sensing and adaptive spatio-temporal modeling.

### Discussion

sEMGCareHCI has explored the potential to identify finger position gestures with the classification algorithm support vector machine (SVM) with emphasis on the classification accuracy of the classifier that uses a small size training data. The focus of this project is to make a contribution to the control technology by using surface electromyography (sEMG) sensors and classification algorithms to effectively forecast the positions of fingers. The development of an embedded device, which combines the sensing and the execution of the special software to perform the prediction, can open up the possibilities of a new kind of human interface device (HID). The preprocessing analysis highlights the critical importance of tailoring signal transformations to the characteristics of individual gesture classes. Robust scaling emerged as the most effective normalization scheme, particularly for gestures with inherently low signal-to-noise ratios. In terms of feature extraction, WL and Hjorth descriptors captured both dynamic and sustained muscle activations, while AR coefficients proved essential for disambiguating push-based gestures. Among time-frequency methods, CWT scalograms demonstrated superior representation of transient directional slides compared to STFT. Finally, autoencoder-based embeddings not only reduced dimensionality but also preserved the underlying nonlinear muscle synergies, thereby enhancing deep sequence model performance. Taken together, the findings indicate that the optimal preprocessing pipeline integrates robust scaling, WL+Hjorth feature extraction, and autoencoder-based dimensionality reduction, forming a strong foundation for the deep-learning architectures presented in the subsequent sections. This pipeline ensures both high classification accuracy and computational feasibility, key requirements for real-time HCI and neuroprosthetic applications. The overlap of some pairs of gestures, as the false positive and false negative rate suggest is a direct consequence of the overlap of the muscle activation pathways. Any pair of gestures needing a similar pattern of activation of the four measured muscles will give similar feature vectors and will be harder to differentiate by the classifier by their very nature. This knowledge gives a strong and obvious explanation of one of the possible directions of future work, which is the incorporation of high-density (HD) sEMG arrays.

## Methods

**Fig. 9:**
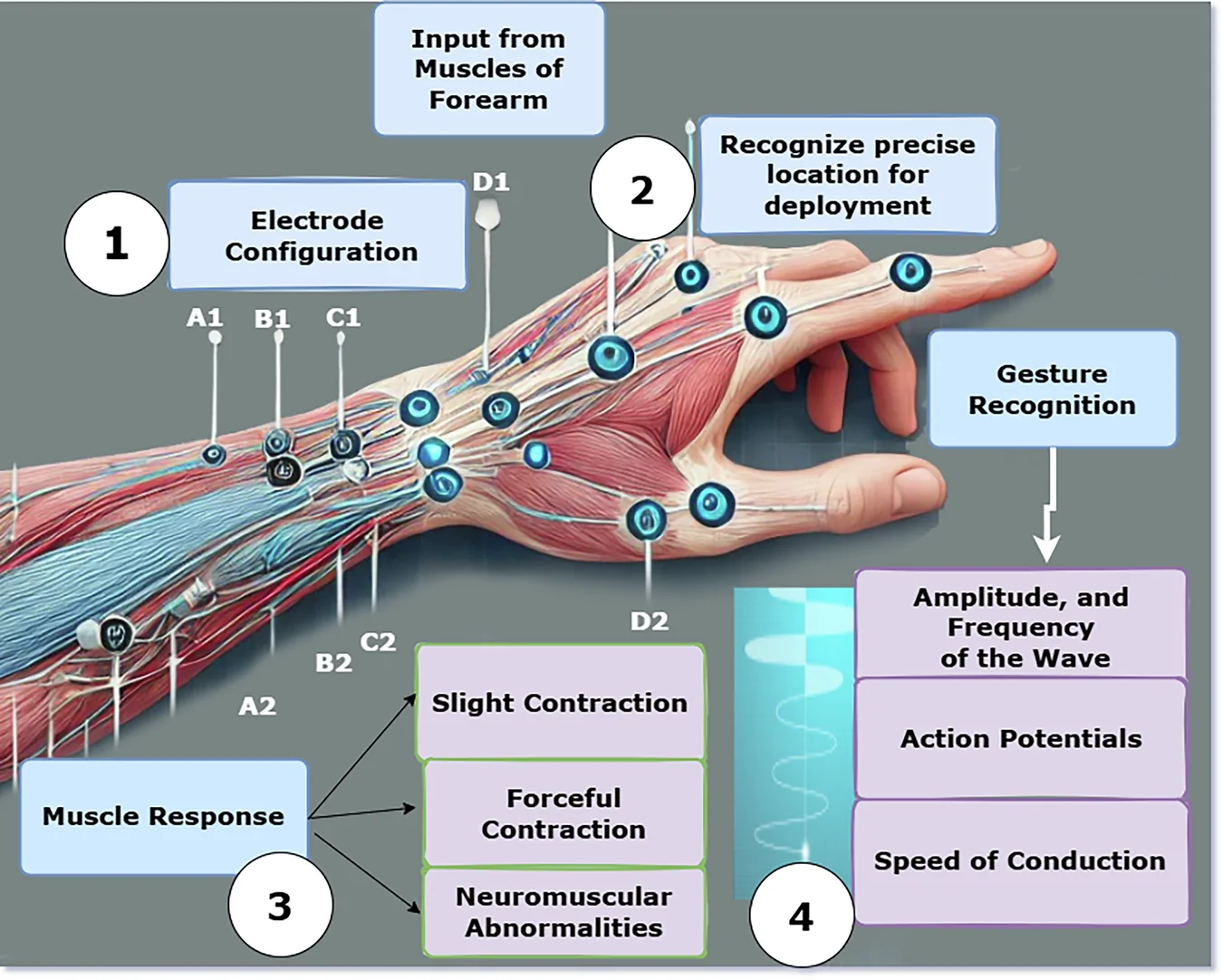
Location on the forearm for sEMG signals to be collected from sensors.

The sEMG sensing unit was devised to get electromyography signals from various locations on the forearm muscles. This is an embedded device that is meant to utilize four generic sEMG sensors to enhance the action potential generated in the targeted muscles of the forearm, as shown in Fig. 9. The sensors A-D are attached to the muscle as described in Table 5. There are three surface electrodes attached to each sensor, Two electrodes are applied to the skin in the perpendicular position of the muscle of interest, one at the middle of the muscle and another at the end of the muscle. The final electrode is a reference electrode, which must be attached to an area of the skin that is distant to the target muscle. The four sEMG sensors are all connected to the same electrode and they have a common reference point. In order to ensure a constant positioning of the electrodes, an armband is made out of an elastic armband and regular EMG lead wires with a socket adaptor that can accept EMG electrodes. Electrodes are attached to the underside of the band on the socket. The electrodes on the armband are set to be located on the surface of the forearm (according to Table 5, the electrodes have a specific position). ECG electrodes are silver/silver chloride (Ag/AgCl) ECG electrodes of a diameter of 20 mm and are single-use and pre-gelled.

**Table 5:** Electrode placement, for superficial muscles of the anterior and posterior forearm

Sensor	Muscle
A	Flexor Carpi Radialis
B	Palmaris Longus
C	Flexor Carpi Ulnaris
D	Extensor Digitorum

Surface EMG signals are inherently noisy due to factors such as electrode-skin impedance, motion artifacts, and interference from external electrical sources. To ensure robust feature extraction and subsequent classification, all recordings underwent a rigorous signal conditioning stage. A fourth-order Butterworth band-pass filter (20–225 Hz), consistent with the Nyquist frequency of the 500 Hz sampling rate, was applied to attenuate both low-frequency drift (movement artifacts and baseline wander) and high-frequency noise (thermal and electronic interference).

Additionally, a notch filter centered at 60 Hz was employed to effectively remove power-line contamination, which is particularly pronounced in biomedical recordings acquired in laboratory and clinical environments. Following filtering, the signals were segmented into overlapping windows to preserve temporal dynamics while reducing computational redundancy. A 250 ms sliding window with 50% overlap was selected after empirical evaluation, providing a balance between temporal resolution (sufficient to capture short, transient gestures such as G4 and G6) and computational efficiency required for real-time HCI applications.

### Signal integrity as a function of amplifier design

**Fig. 10:**
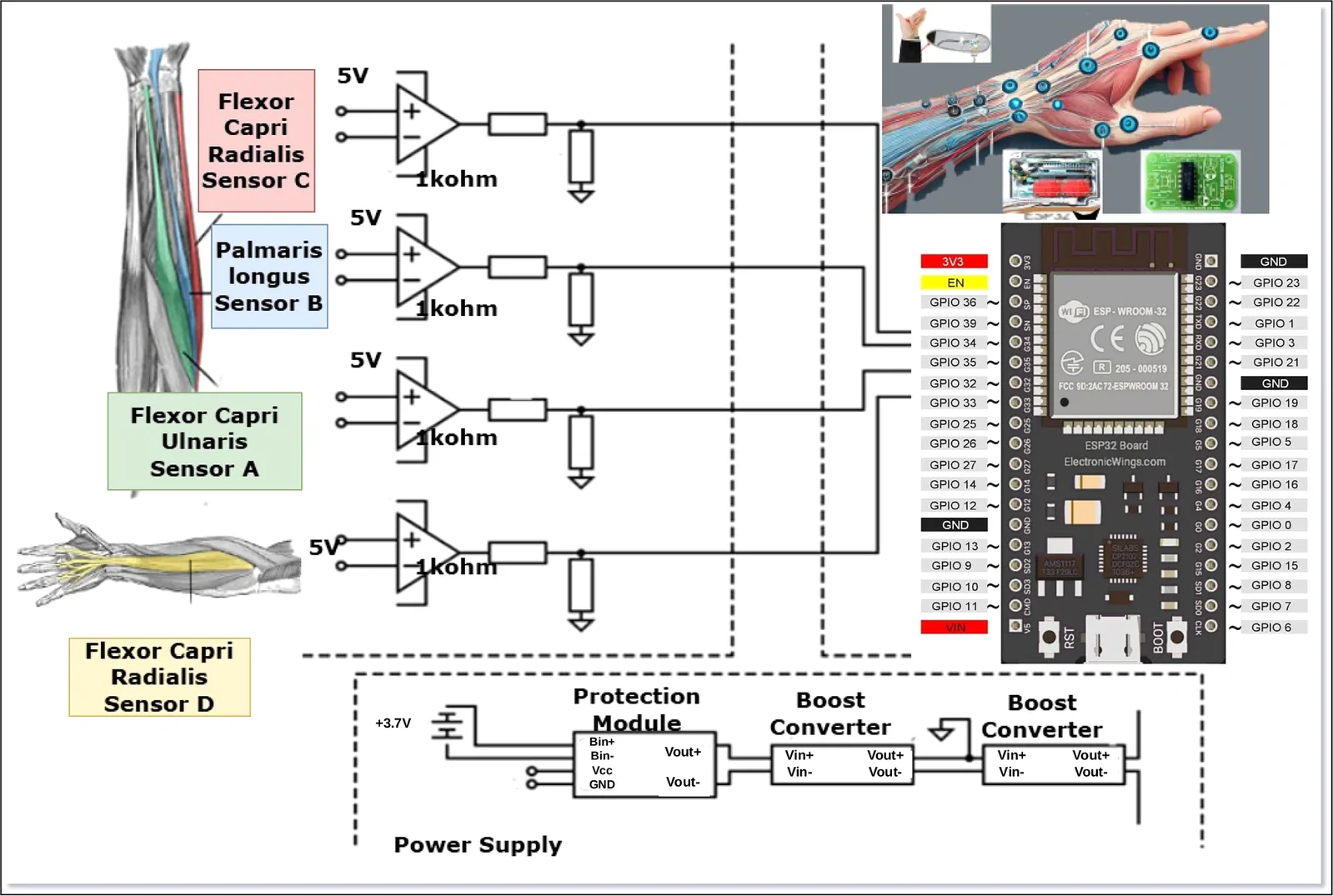
sEMG sensing unit—hardware (left: armband, right: device).

The sEMG signal on the skin surface is a small bio-potential having amplitudes of several microvolts to several millivolts. This weak signal is buried in a sea of environmental noise, the most significant being the 50/60 Hz power-line interference, which can be many orders of magnitude stronger. The most critical step towards ensuring that a usable signal is obtained is therefore the analog front end of the sEMG sensing unit. The design that this work proposes uses a complex, bespoke circuit to overcome this obstacle head-on. sEMGCareHCI describes how to implement the hardware description from each sensor to the boost converter in Fig. 10 (in both hardware and software design). The software entails the creation of the embedded system, which handles the capture of the EMG signals and the execution of filtering and processing of the signals. Lastly, the creation of the desktop program that performed the classification and prediction of the finger position of the processed signal was based on machine learning. The two-stage amplification strategy is a pillar of this design. Any more amplification would run the risk of saturating the amplifier and cutting off the signal peaks, as well as permanently destroying information. This is avoided by the implemented two-stage approach, which uses an AD621 instrumentation amplifier to offer a fixed initial gain of 100 in the first stage and an OP07 operational amplifier to offer the rest of the gain of 20 in the second stage. This cascaded structure makes sure that the signal is boosted to a size that can be converted to analog-to-digital conversion without distortion. The deployment of AD621 as the first-stage instrumentation amplifier is a design choice that is the most critical and motivated by its extraordinary performance characteristics. The most significant of them is its high Common-Mode Rejection Ratio (CMRR), which is stated to be around 130 dB^15^. CMRR is a measure of how well an amplifier can reject simultaneous signals that are in phase on both of its inputs (i.e., common-mode signals) and amplify the signal that is not common between the two inputs (i.e., the differential signal). The fact that the two differential electrodes on the skin detect almost equal levels of environmental noise such as power-line interference results in it appearing as a large common-mode voltage. A rejection factor of more than 3,160,000:1 is equivalent to a CMRR of 130 dB. This implies that the amplifier will reduce the common-mode noise by more than three million times and will amplify the intended differential sEMG signal. This phenomenal rejection is the main defense of the system against external noise and the basis of deriving a clean microvolt-level physiological signal out of a noisy environment.

Besides the external artifacts, there is also internally generated noise caused by the power supply that may contaminate the signal. A lithium-ion battery is used to power the system, controlled by boost converters and may cause high-frequency switching ripple on the supply rails. The Power Supply Rejection Ratio (PSRR) of the amplifier is the measure of the capacity to reject this noise. AD621 (PSRR of 140 dB) and the OP07 (PSRR of 5 μV) were selected because of their high PSRR. This is further enhanced with the addition of several 220 μF electrolytic capacitors into the power supply outputs and that these serve as local charge reservoirs to eliminate high-frequency ripple. This is a care taken to ensure that the power supply of the amplifier does not become a source of signal contamination in its own right.

These design features are not independent optimizations, which are two-stage gain, high CMRR, high PSRR, and accurate filtering. They are a synergistic mechanism that is designed to optimize the signal-to-noise ratio (SNR) of sEMG signal long before it enters the digital world. It is these thoughtful and justified bio-instrumentation design decisions upon which the high classification performance is constructed and is a direct consequence of them.

### Fidelity of digital conversion and real-time processing

The next important stage of the data acquisition pipeline is the conversion of the clean, amplified analog signal to a digital frequency. The quality of the Analog-to-Digital Converter (ADC) and the security of the timing of the data acquisition have a direct effect on the quality of the data that can be used by machine learning. Lastly, the analog front-end includes a well-defined band-pass filter in the passband of 3 Hz to 400 Hz. 3 Hz to 400 Hz refers to the analog filter, and 20–450 Hz refers to the digital post-processing filter, analog (AFE, 3–400 Hz, pre-ADC, anti-aliasing) and digital (DSP, 20–450 Hz, post-ADC) filter stages. The 450 Hz upper cut-off of the digital band-pass filter is a filter design parameter; it does not imply that signal energy above the Nyquist frequency (250 Hz at *f*_*s*_ = 500 Hz) is present in the digitised data. Anti-aliasing is ensured by the Stage 1 analog low-pass roll-off centered at 400 Hz, which attenuates all components above *f*_*N*_ before they reach the ADC. This has two purposes: the 3 Hz high-pass filter is an effective removal of the very low-frequency (quasi-DC) baseline drift that occurs due to motion artifacts, which is produced by motion between the electrode and the skin. The 400 Hz low-pass filter removes any irrelevant high-frequency noise and, more importantly, is an anti-aliasing filter. This allows such higher frequencies not to enter the ADC and thus not be folded back into the measurement band incorrectly, corrupting the signal, by making sure that frequencies above the Nyquist frequency (250 Hz at a 500 Hz sampling rate) do not reach the ADC. The sampling rate used in the system is 500 Hz, which is sufficiently suited to the physiological characteristics of sEMG signal. Most of the usable energy of sEMG signal is concentrated in the 20–400 Hz frequency band, with the most dominant frequencies usually falling between 50 Hz and 150 Hz^5^. Although this implies that some of the higher-frequency signal material (250–400 Hz) is not recorded, it is adequate to record the most vigorous and informative part of the signal spectrum. Here, the low-pass anti-aliasing filter at 400 Hz in the analog front-end is important because it avoids aliasing distortion corrupting the sub-250 Hz band and makes the sampled data valid.

The ESP32 microcontroller’s internal 12-bit ADC digitizes the signal. Although a nominal 12-bit resolution allows, in theory, 4096 different quantization levels, it is generally accepted that the on-chip ADCs of general-purpose microcontrollers have severe non-linearity and noise, especially at the ends of their input voltage range. A more realistic measure of converter performance, which is usually denoted by the Effective Number of Bits (ENOB), is the actual converter resolution, which is nearly always less than the nominal converter resolution. The ENOB is estimated to be in practice between 8 and 9 bits, rather than 12 (an ESP32). It implies that the ADC itself will provide a non-trivial source of quantization error and noise, which will be a performance bottleneck in the signal chain. This is a major design compromise, in which the simplicity and low cost of an internal ADC are compromised against the greater fidelity of an external ADC component^3^.

The operational stability of any interactive system should be guaranteed in real time. An interactive HCI demands that information be received and processed and responded to with an extremely low latency. It uses the FreeRTOS (Real-Time Operating System) that is already on the ESP32 to have two simultaneous time-out functions running, one to read the analog signal and the other to send the digitized data out of the serial port. The 500 Hz sampling is implemented with a minimum amount of time jitter, which otherwise could be induced by blocking function calls or simple polling loops in a non-real-time architecture. This deterministic time is necessary to the stability of the next feature extraction and classification algorithms, which require a constant data rate.

The presence of the ADC as a performance bottleneck offers an important insight into the system performance in general. This is despite the limitations and noise added at the digital conversion stage, which does not affect the high classification accuracy. This heavily suggests two things: firstly, that the signal quality provided by the custom analog front-end is of very high quality, and this gives a robust signal that can be slightly degraded. Second, it indicates that the selected feature (Waveform Length) and classifier (RBF-SVM) are incredibly robust and can be able to peep through the noise and non-linearity caused by ADC, and retrieve the discriminatory patterns embedded in the data. This strength is a tribute to the strength of the software pipeline, yet it also obviously points to the most effective direction of future hardware update, the addition of an external, high-ENOB (e.g., 16-bit or 24-bit) delta-sigma ADC to maximize the benefits of the clean signal delivered by the analog front-end^1^.

**Fig. 11:**
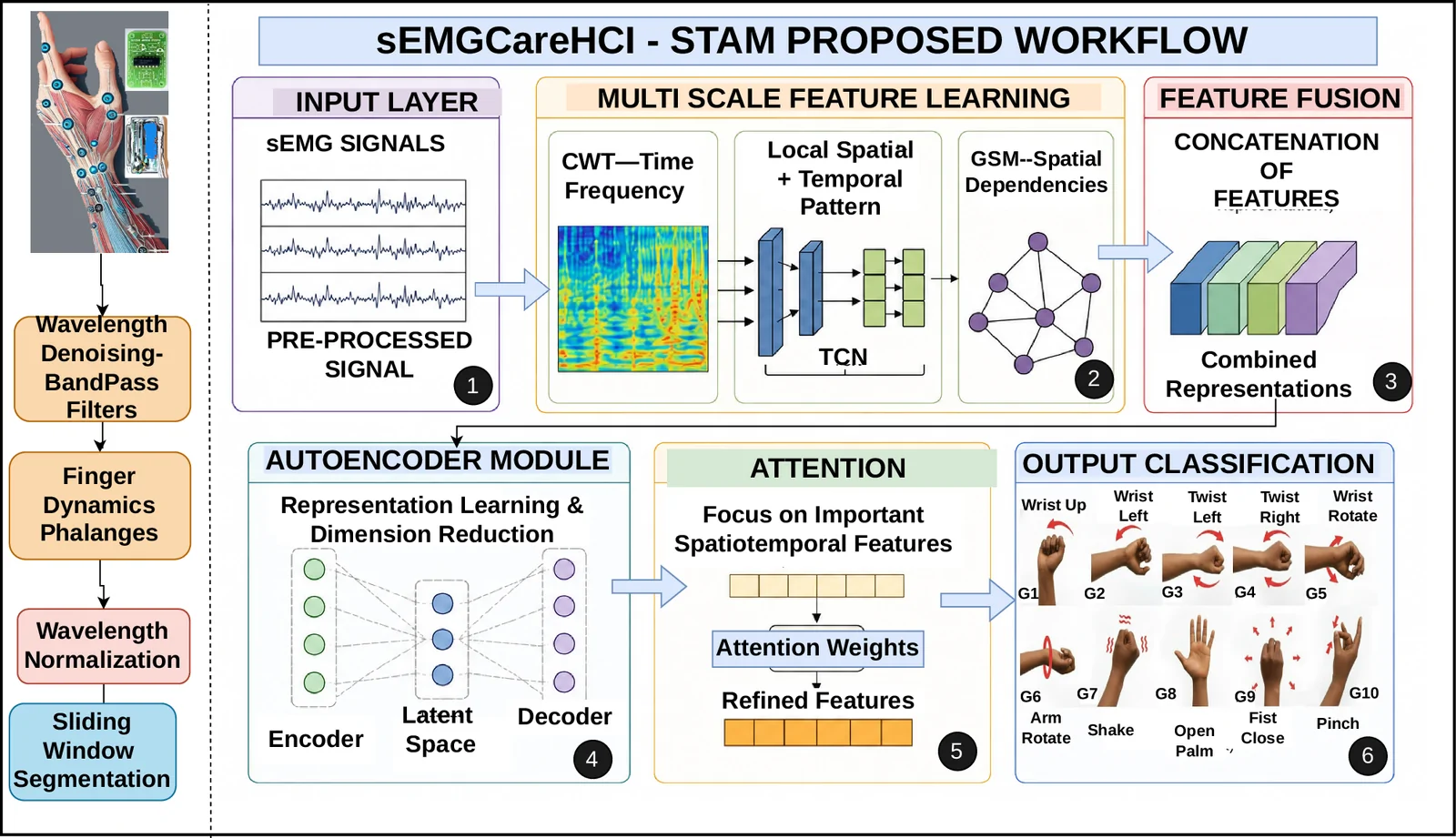
sEMGCareHCI: block diagram.

Making bio-potentials translatable into trusted digital instructions is the keynote of any useful electromyography-based human-computer interface (HCI). The accuracy of the machine learning classifier is not only the final value but also an overall measure of the system’s effectiveness, from signal acquisition to feature representation. This part gives a quantitative breakdown of the results of the gesture classifications, providing the empirical basis for a further discussion of the biomechanical and bio-instrumentation principles underlying system functionality. The results are analyzed in terms of three major factors, namely, the reason why the selected classifier succeeded, the discriminative power of the chosen signal feature, and the physiological explanation of the difference in performance based ongestures sEMGCareHCI-STAM Proposed workflow, as in Fig. 11. Correlating Lagrangian Dynamics with sEMG Feature Magnitude: The biomechanical analysis presents a sophisticated model of the finger’s dynamics using the Lagrangian formulation. The resulting Euler-Lagrange equation of motion, given by

1 $$\frac{d}{dt}\left(\frac{\partial L}{\partial {\dot{Q}}_{j}}\right)-\frac{\partial L}{\partial {Q}_{j}}+{f}_{{\rm{friction}}}={\tau }_{j}$$

Equation (1) is a powerful tool. It allows for the calculation of the net joint torque (*τ*_*j*_) required to execute a specific gesture, accounting for the inertial properties of the finger segments, gravitational forces, and friction. This theoretical torque provides a direct link to the empirical sEMG measurements. One of the foundations of biomechanics is the direct relationship between the electrical activity recorded by the sEMG and the force and, consequently, the torque generated by the underlying muscle. Increasing the central nervous system is necessary to produce a larger torque by recruiting additional motor units and/or increasing the rate of firing, which leads to a larger-amplitude, more complicated sEMG signal. This creates a very definite chain of causation: the biomechanical model forecasts the amount of torque necessary, and the sEMG signal indicates the amount of muscular effort to generate that amount of torque.

### Kinematic velocity and acceleration as proxies for signal non-stationarity

In addition to the stationary forces, the biomechanical model can also give a complete kinematic description of the motion, including the velocity of each phalanx ($b{\dot{y}}_{j}^{p}$, $b{\dot{z}}_{j}^{p}$), and, by differentiation, the acceleration of each phalanx. Such kinematic quantities are also important since the relationship between muscle force and the resulting sEMG signal is not linear; it is strongly modulated by the dynamics of the contraction. Spectral content and non-stationarity properties of sEMG signal vary greatly with whether the contraction of a muscle is isometric (static, no length change), concentric (shortening under load), or eccentric (lengthening under load) and the speed of such change. Rapidly performed gestures, which are associated with high velocity and acceleration variables in the kinematic model, will result in more clearly non-stationary sEMG signals. The statistical characteristics of the signal will rapidly vary in the analysis window and present an increased challenge to feature extraction.

The control trajectory of the model $T=A\sin (3(t-{t}_{0}))$ gives a given dynamic profile to the simulated movements. This trajectory is sinusoidal in nature; that is, the velocity does not always have the same value. At the beginning and the end points of the motion, the velocity is zero and in the middle, the velocity is the greatest. The amplitude and frequency content of the sEMG signal produced by the muscles that perform the movement will be directly modulated by this time-varying velocity profile. The regular success of the Waveform Length (WL) feature can be partially explained by the fact that the latter is capable of capturing these dynamic changes as well. WL is a good fit to determine the complexity of these non-stationary signals generated during dynamic contractions as a measure of the cumulative rate of change of the signal. This combination of kinematics and sEMG gives a deeper insight into what the classification system actually attains. It is not just that the system is differentiating the abstract gesture labels such as “hand open” and “hand close”. It is separating the individual, time-varying, multi-channel distributions of muscle activity that give the particular torques, velocities, and acceleration determined by the biomechanics of each gesture. The job of the classifier is to reverse trace the electrical manifestation measured to the causal gesture. Thus, a gesture can be broken down into its basic physical elements using the biomechanical model. The classification performance is made to be a factor of the capability of the system to resolve the disparity in these underlying physical components as reflected by the sEMG sensors. This gives the system a very technical and physically based interpretation of the system’s functionality and its performance constraints.

By pursuing these integrated software and hardware advancements, the foundational work presented here can be extended to create a new generation of sEMG-based interfaces that are not only accurate but also robust, generalizable, and practical for real-world assistive applications.

**Fig. 12:**
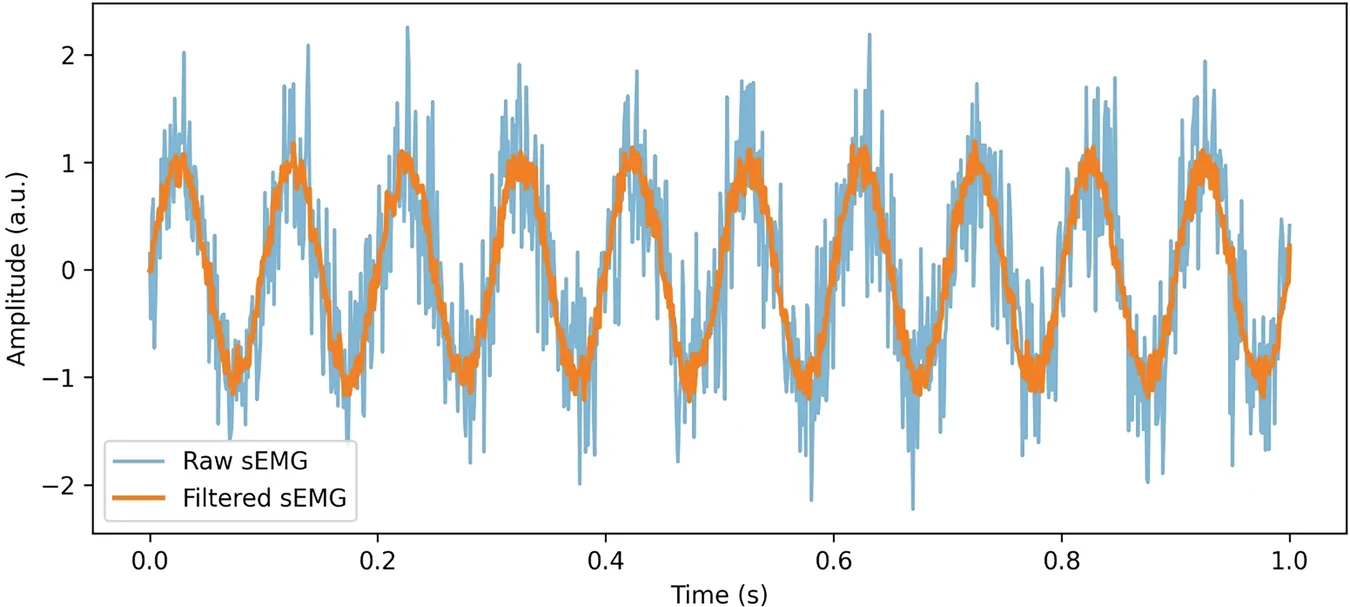
Raw and filtered signals.

Figure 12 illustrates an example of raw and filtered signals for the middle pinch gesture. The raw trace demonstrates significant baseline drift and superimposed noise, whereas the filtered signal reveals clean and distinct activation bursts corresponding to finger contractions, confirming the effectiveness of the preprocessing pipeline.

Due to subject-specific differences in muscle physiology, electrode placement, and contraction force, normalization plays a crucial role in ensuring model generalization. We systematically evaluated three widely used scaling methods: 1. Z-Score Normalization: standardizes signals to zero mean and unit variance, mitigating inter-channel differences but sensitive to outliers. 2. Robust Scaling: centers signals around the median and scales by the inter quartile range, reducing the influence of extreme values. 3. Min–Max Scaling: linearly scales amplitudes into the [0,1] range, widely used for neural networks but prone to distortions in the presence of outliers.

### Experimentation study

The sEMGCareHCI involves four sensor channels being placed on the particular muscles of the forearm: Flexor Carpi Radialis (FCR), Palmaris Longus (PL), Flexor Carpi Ulnaris (FCU), and Extensor Digitorum (ED). Strong, decisive contractions of these large groups of muscles can result in high-amplitude, clear sEMG patterns that can be easily separated by the classifier. As an example, extensor Digitorum is massively involved in the movement of the little finger extension (LFE) and the flexor group (FCR, FCU, PL) is heavily involved in the movement of the wrist flexion (WF). These gross motor actions produce strong, comparatively clean signals, which produce feature vectors occupying non-overlapping and remote regions in the feature space, which causes high classification accuracy. Data were collected from 8 healthy participants and 10 subjects between 18 and 40 years old having fingers amputated or non-functioning. Amputee/non-functioning-finger status was confirmed through participant self-report and visual/clinical assessment at the recruitment site, and participants with residual forearm muscle activity sufficient to produce a detectable sEMG signal at rest and during attempted gesture execution were included; individuals with skin conditions precluding electrode adhesion or with insufficient residual muscle activity were excluded. All participants provided informed consent prior to data acquisition. The healthy participant group was included to establish baseline gesture separability under standard neuromuscular conditions, whereas the amputee/non-functioning-finger group represented the primary target population for the proposed amputee-centric HCI framework.

Each data collection session followed a fixed protocol: the armband was fitted and electrode placement was verified against the locations in Table 5, followed by a short calibration period in which the participant performed each of the 10 gestures (G1–G10) once under visual guidance to confirm signal quality. Data acquisition then proceeded with 5 cued repetitions per gesture, with gestures cued visually at a fixed pace and a brief rest interval between repetitions to minimize fatigue and motor adaptation effects, yielding 50 labeled samples per participant as described above.

The deployed system used real-time testing to validate the trained classifier, which was embedded in the HCI system. The system processed incoming sEMG signals using a sliding window technique that operated for 250 ms with 50% overlap. Each segmented window was assigned the corresponding gesture label and used for feature extraction and model evaluation. The system produced continuous gesture predictions while accuracy measurement occurred during a live session that lasted for 12 min per subject. To improve temporal stability and reduce isolated misclassifications during real-time deployment, majority voting was applied across consecutive prediction windows. The authors are thankful to the “Vishal Neurodiagnostic Center”, Gujarat, India, for clearance from the ethical committee with reference number VISD/RG/020825/ETH106 to collect the sEMG samples from the subjects.

.

### Group-wise performance analysis

In the present study, healthy participants and amputee/non-functioning-finger subjects were pooled during model development and evaluation because the primary objective was to learn gesture-specific sEMG activation patterns across heterogeneous users, rather than to compare the two participant groups. Both groups followed the same sensor placement, acquisition protocol, preprocessing pipeline, sliding-window segmentation, feature extraction, and gesture-labeling procedure. Pooling the data increased inter-subject variability and exposed the model to a broader range of neuromuscular signal characteristics, which is important for improving robustness in real-time HCI applications. However, we acknowledge that the present study does not report separate cohort-specific performance for healthy and amputee/non-functioning-finger subjects. This has been added as a limitation, and future work with a larger cohort will perform dedicated subgroup analysis to quantify performance differences between participant groups. The system used majority voting across three consecutive windows to enhance temporal stability during its deployment process.

**Fig. 13:**
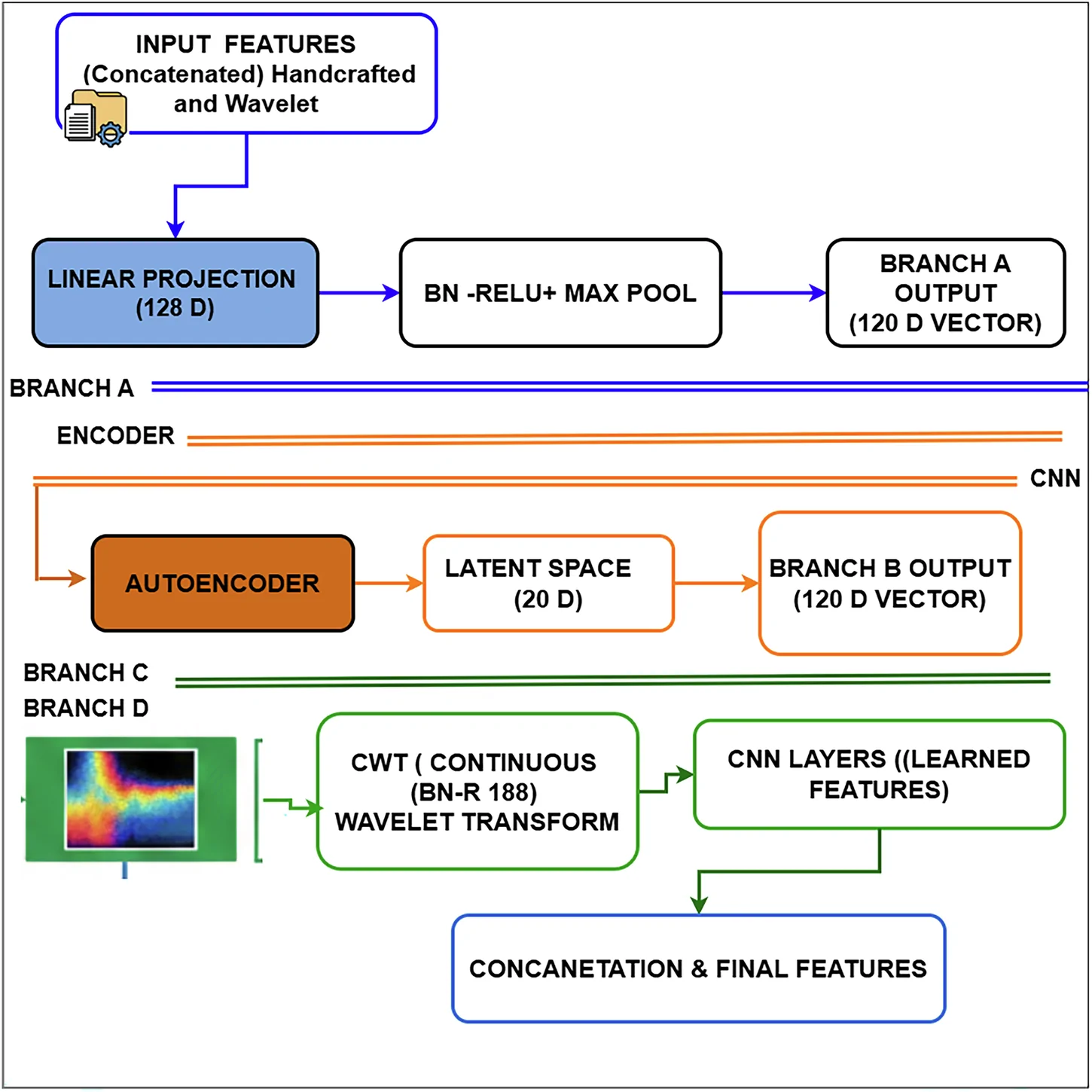
Concatenation of final features.

**Fig. 14:**
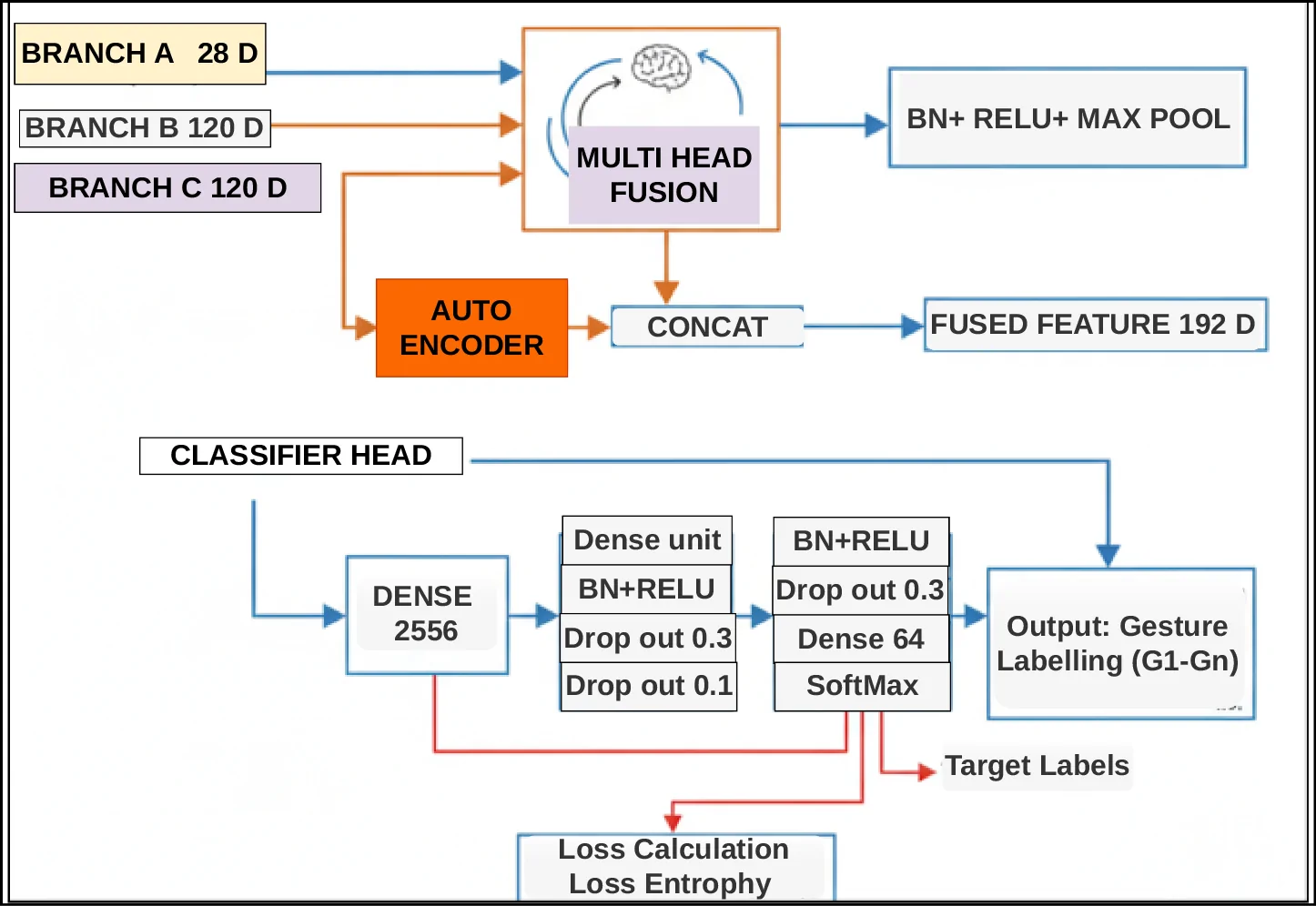
Multi-head attention fusion.

**Fig. 15:**
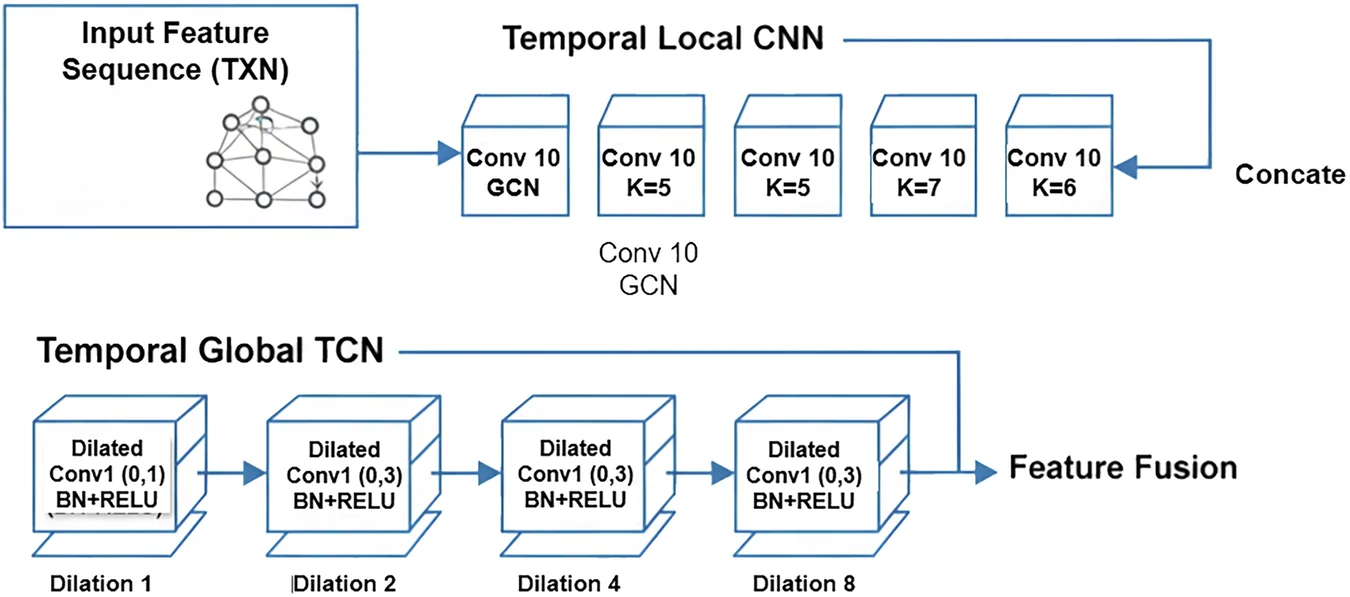
Sequence of input features.

Figure 13 explains the concatenation of final features for the representations of a G1 gesture, where CWT demonstrates clearer, temporally localized frequency bursts. Given the high dimensionality of feature sets (120 features per window), dimensionality reduction was explored to enhance model efficiency and stability: Principal Component Analysis (PCA) reduced features to 30 components, retaining 95% of variance while lowering computational overhead. t-Distributed Stochastic Neighbor Embedding (t-SNE) is employed for visualization, projecting high-dimensional features into 2D clusters to assess separability. Autoencoder-based Embeddings: nonlinear reduction to a compact 20-dimensional latent space, learned directly from sEMG data. PCA improved training stability for Transformer-based models, reducing convergence time by approximately 25%. Autoencoder embeddings enhanced CNN-BiLSTM macro-F1 by 2.3%, Indicating their capacity to capture nonlinear synergies across muscle activations. Handcrafted features (WL/Hjorth) → Dense layer (128 units, ReLU). Autoencoder embeddings → Dense layer (128 units, ReLU). Scalograms → Convolutional stack (Conv2D → BN → ReLU → MaxPool), producing a 128-dimensional feature map. All three branches are projected into a shared 128-D embedding space, forming a harmonized representation suitable for multimodal fusion, as in Fig. 14. We propose the Spatio-Temporal Attention Model (STAM), a hybrid multi-branch deep-learning architecture that integrates heterogeneous sEMG preprocessing outputs into a unified framework for robust gesture recognition as in Fig. 15. Instead of relying on a single input type, STAM assigns dedicated branches to handcrafted statistical features (WL and Hjorth), autoencoder latent embeddings, and CWT scalograms, projecting them into a shared embedding space for multimodal fusion. Spatial dependencies among electrodes are modeled using a Graph Convolutional Network (GCN), capturing inter-electrode relationships based on anatomical proximity and signal correlation. Temporal dynamics are learned through a CNN-TCN hybrid module, effectively modeling both short-term muscle activations and long-range dependencies. An attention-based fusion mechanism adaptively re-weights spatial, temporal, and frequency features according to gesture type, producing a discriminative fused representation. Finally, a regularized dense classification head maps the fused features to 10 HCI-2 gesture classes, ensuring accurate and generalized predictions.

## Data Availability

The datasets utilized in this research for the purpose of training and evaluating for the proposed models will be submitted on request.
